# Anemia, iron status, and associated protective and risk factors among children and adolescents aged 3 to 19 years old from four First Nations communities in Quebec

**DOI:** 10.17269/s41997-020-00304-7

**Published:** 2020-03-13

**Authors:** Emad Tahir, Pierre Ayotte, Matthew Little, Richard E. Bélanger, Michel Lucas, Donna Mergler, Elhadji A. Laouan Sidi, Community of Winneway - Long Point First Nation, Community of Lac Simon, CSSS Tshukuminu Kanani of Nutashkuan, Community of Unamen Shipu, Nancy Gros-Louis McHugh, Mélanie Lemire

**Affiliations:** 1 Département de médecine sociale et préventive, Université Laval, Quebec City, QC Canada; 2 Axe santé des populations et pratiques optimales en santé, Centre de recherche du CHU de Québec, Université Laval, Quebec City, QC Canada; 3 Centre de toxicologie du Québec, INSPQ, Quebec City, Canada; 4 School of Public Health and Social Policy, University of Victoria, Victoria, Canada; 5 Département de pédiatrie, Université Laval, Quebec City, QC Canada; 6 CINBIOSE, Université du Québec à Montréal, Montreal, QC Canada; 7 First Nations of Québec and Labrador Health and Social Services Commission, Wendake, QC Canada; 8 Centre de Recherche du CHU de Québec, Hôpital du Saint-Sacrement, 1050 chemin Sainte-Foy, Quebec City, QC G1S 4L8 Canada

**Keywords:** Childhood anemia, Iron deficiency, First Nations, Vitamin C, Inflammation, Anémie pédiatrique, Carence en fer, Premières Nations, Vitamine C, Inflammation

## Abstract

**Objectives:**

Anemia and iron deficiency (ID) are frequent among Indigenous children of Canada, but few data are available in Quebec. The present study aimed to characterize anemia and ID prevalence and associated protective and risk factors among First Nations youth in Quebec.

**Methods:**

The 2015 First Nations (JES!-YEH!) pilot study was conducted among children and adolescents (3 to 19 years; *n* = 198) from four First Nations communities in Quebec. Blood and urine samples and anthropometric measurements were collected. Hemoglobin (Hb), serum ferritin (SF), plasma hs-CRP, and urinary cotinine levels were measured. Factors associated with anemia and ID (including traditional and market food consumption) were assessed using an interview-administered food frequency questionnaire, based on which nutritional intakes were calculated. Structural equation models were used to test associations.

**Results:**

The prevalence of anemia and ID was elevated (16.8% and 20.5% respectively). Traditional meat, fruit, and fruit juice (natural and powdered)—via their positive association with vitamin C intake—were the only food variables positively associated with SF (coefficient [95% CI] 0.017 [0.000, 0.114]; 0.090 [0.027, 0.161]; and 0.237 [0.060, 0.411]). Male sex was also associated with higher SF (0.295 [0.093, 0.502]). Inflammation status (hs-CRP > 5 mg/L) was inversely associated with Hb (− 0.015 [− 0.025, − 0.005]), whereas SF was positively associated with Hb (0.066 [0.040, 0.096]). Fruit and juice consumption was also positively associated with Hb, via vitamin C intake and SF (0.004 [0.001, 0.010]; 0.008 [0.003, 0.017]).

**Conclusions:**

Interventions fostering healthier food environments as well as higher consumption of traditional meats and foods naturally rich in vitamin C, which is known to enhance iron absorption, and fighting inflammation could contribute to decrease the high prevalence of anemia and ID in this young Indigenous population.

**Electronic supplementary material:**

The online version of this article (10.17269/s41997-020-00304-7) contains supplementary material, which is available to authorized users.

## Résumé

**Objectifs:**

L’anémie et la carence en fer sont fréquentes chez les enfants autochtones au Canada, mais peu de données sont disponibles au Québec. La présente étude visait à caractériser la prévalence de l’anémie et de la carence en fer et les facteurs protecteurs et des risques associés chez des jeunes des Premières Nations du Québec.

**Méthodes:**

En 2015, l’étude pilote Jeunes Environnement et Santé (JES! - YEH!) a été menée chez des enfants et des adolescents (3 à 19 ans, *n* = 198) de quatre communautés de Premières Nations au Québec. Des échantillons de sang et d’urine et des mesures anthropométriques ont été recueillis. Les concentrations d’hémoglobine, de ferritine sérique, de hs-CRP plasmatique et de cotinine urinaire ont été mesurées. Les facteurs associés à l’anémie et à la carence en fer (y compris la consommation d’aliments traditionnels et de marché) ont été évalués à l’aide de questionnaires de fréquences alimentaires administré par un assistant de recherche, à partir desquels les apports nutritionnels ont été calculés. Des modèles d’équations structurelles ont été utilisés pour tester les associations.

**Résultats:**

La prévalence de l’anémie et de la carence en fer était élevée (16,8 % et 20,5 % respectivement). La viande traditionnelle, les fruits et les jus de fruits (naturels et en poudre) − par l’intermédiaire de leur association positive avec l’apport en vitamine C − étaient les seules variables alimentaires positivement associées à la ferritine sérique (coefficient [IC à 95 %] : 0,017 [0,000, 0,114]; 0,090 [0,027, 0,161]; et 0,237 [0,060, 0,411]). Le sexe masculin était également associé à une ferritine sérique plus élevée (0,295 [0,093, 0,502]). Le statut inflammatoire (hs-CRP > 5 mg/L) était inversement associé à l’hémoglobine (− 0,015 [− 0,025, − 0,005]) alors que la ferritine sérique était positivement associée à l’hémoglobine (0,066 [0,040, 0,096]). La consommation de fruits et de jus était aussi positivement associée à l’hémoglobine via l’apport en vitamine C et la ferritine sérique (0,004 [0,001, 0,010]; 0,008 [0,003, 0,017]).

**Conclusions:**

Les interventions favorisant des environnements alimentaires plus sains ainsi qu’une consommation plus élevée de viandes et d’aliments traditionnels naturellement riches en vitamine C, qui sont connus d’améliorer l’absorption de fer, ainsi que luttant contre l’inflammation pourraient contribuer à diminuer la prévalence élevée d’anémie et de la carence en fer dans cette jeune population autochtone.

## Introduction

Canada is among countries with the lowest prevalence of childhood anemia. In 2009–2011, 97% to 99% of Canadian children and adolescents had sufficient hemoglobin (Hb) levels, which means that less than 3% of the Canadian youth general population presented anemia (Cooper et al. 2012). However, anemia prevalence is invariably higher among Indigenous populations all over North America (Christofides et al. 2005; Jamieson et al. 2016). Individuals with mild to moderate forms of anemia are physically limited by the fatigability, shortness of breath, dizziness, and muscle weakness due to inadequate tissue supply of oxygen. However, those with severe cases of anemia can progress to heart failure and even death (Turkoski 2003).

Indigenous populations in Canada refer to First Nations, Métis, and Inuit Peoples. They have lived in what is now Canada long before the arrival of Europeans and now make up 5% of the national population (Statistics Canada 2017). First Nations are the largest group (65%), with a relatively younger population when compared with non-Indigenous Canadians (Kelly-Scott and Smith 2015). Each First Nation is composed of many communities that share common language, culture, and traditional diet (Chan et al. 2019). In these communities, dietary shifting from traditional foods, largely composed of wild animal, fish, birds, fruits, and plants, to poor-quality market foods lower in iron often results in iron deficiency (ID) (INSPQ 2015) and if severe enough to impair erythropoiesis, to iron deficiency anemia (IDA) (Christofides et al. 2005). Furthermore, inflammation secondary to infection and obesity disturbs Hb and red blood cell synthesis, and results in anemia of chronic inflammation (ACI) (Jamieson et al. 2016; Cash and Sears 1989). Other vitamin deficiencies, along with lead (Pb) intoxication (at exposure levels above 100 μg/L), are also known causes of anemia that some researchers in population studies regroup as unexplained anemia (UA) (Christofides et al. 2005; Jamieson et al. 2012; Plante et al. 2011; Hegazy et al. 2010). Other possible causes of anemia are hemorrhagic, hemolytic, and anemia linked to hemoglobinopathies, although they are all less common in First Nation contexts (Jamieson and Kuhnlein 2008).

Worldwide, ID is the most common nutritional deficiency (de Benoist et al. 2008; WHO 2001). ID has detrimental side effects such as reduced memory and attention, defective thermoregulatory mechanisms, and defective immunity (Lopez et al. 2016). It may occur directly from inadequate iron intake, or indirectly due to reduced intestinal absorption rate of iron. Contrary to iron from animal meats (heme iron) that has superior bioavailability, the intestinal absorption of non-heme iron, found in fruits, vegetables, and cereals, is influenced positively by increased gastric acidity and concurrent intake of iron-absorption enhancers (vitamin C, vitamin A, and proteins from animal meats) (WHO 2001). Conversely, the intake of iron-absorption inhibitors (dairy products, tea, egg protein, etc.) prevents non-heme iron absorption (WHO 2001). Moreover, bacterial and parasitic infections such as *Helicobacter pylori*, which is common in regions facing precarious housing conditions and poor hygiene, reduce gastric acidity and then decrease non-heme iron absorption. Also, *H*. *pylori* infection further contributes to ID as a result of intestinal bleeding (Jamieson and Kuhnlein 2008).

Data on anemia and ID prevalence are scarce in First Nations youth in Canada as First Nations populations are not included in the Canadian Health Measures Survey (CHMS) (St-Amand 2017). Moreover, the very few existing studies on anemia and ID among First Nations are limited to preschool children and adult populations (Christofides et al. 2005). Thus, the present study aimed to document the prevalence of anemia and ID and their associations with possible protective and risk factors among children and adolescents (3–19 years) from two Anishinabe and two Innu communities in Quebec.

## Materials and methods

### Study population

**Fig. 1:**
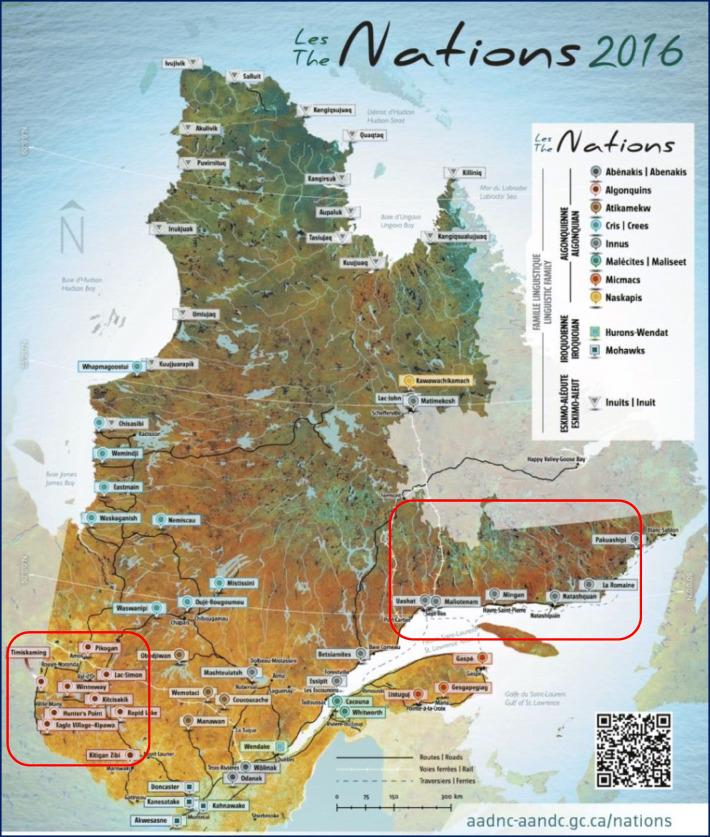
Map of the Quebec province. The Abitibi-Témiscamingue (left) and Minganie and Lower-North-Shore (right) regions where the study took place are indicated by red rectangles. (For interpretation of the references to colour in this figure legend, the reader is referred to the web version of this article)

The *Jeunes, Environnement et Santé*/Youth, Environment and Health (JES!-YEH!) pilot project is a cross-sectional study realized in 2015 in collaboration with two Innu and two Anishinabe First Nations communities that involved 198 participants aged 3 to 19 years old. The aim of the JES!-YEH! pilot study was to document exposure to environmental contaminants, nutritional and health status, and other health determinants in First Nation children and young adults. Further details can be found in Caron-Beaudoin et al. (2019). The two First Nations targeted for the study were selected in collaboration with study partners at the First Nations of Quebec and Labrador Health and Social Services Commission (FNQLHSSC), primarily to represent two distinct ecological regions of the Quebec province (Fig. 1). The Abitibi-Témiscamingue is an inland region with multiple lakes in Northwest Quebec. It is the natural habitat of several wildlife species, such as freshwater fish, moose, beaver, and black bears, and a region characterized by several mining, forestry, and agricultural activities. The Minganie and Lower-North-Shore comprise a large coastal region in Northeast Quebec, where fisheries are the central community and economic activities. All Anishinabe and Innu communities from these two regions with > 500 children and youth were invited to participate. The four communities participating in the project were involved primarily based on their interest in the pilot study. Field research periods (May and June 2015 for Anishinabe communities and September and October 2015 for Innu communities) were selected with community partners to not interfere with hunting and fishing periods as well as other important local events.

This study was approved by the ethical boards of the *CHU de Québec-Université Laval* (no. C14-08-2105) and Health Canada (no. 2014-0043). Community leaders signed a community research agreement, inspired by the research protocol of First Nations of Quebec and Labrador and respecting the OCAP® (ownership, control, access, and possession) principles (Basile et al. 2014). Communities and FNQLHSSC partners were involved at all steps of the study, including the design, data collection, data interpretation, and presentation of results in the communities and at the regional level, as well as co-authors of the present study. Participants’ results for anemia, ID, and other health measurements detailed in Table 1 were returned in person to the participants’ parents or legal guardians, and local clinical follow-ups and interventions were undertaken when relevant.

**Table 1:** Characteristics of all study participants and by nation

Variables	All participants (*n* = 191)		Anishinabe (*n* = 107)		Innu (*n* = 84)	
	*n*	GM [min-max]	*n*	GM [min-max]	*n*	GM [min-max]
Continuous variables						
Age (years)^a^	191	10.3 [3, 19]	107	9.8 [3, 19]	84	10.6 [3, 19]
BMI	189	24.0 [14.3, 64.7]	107	20.7 [14.3, 37.8]	82	26.5 [16.1, 64.7]**
Hb (g/L)	191	125.7 [60.0, 175.0]	107	124.6 [88.0, 166.0]	84	125.3 [60.0, 175.0]
SF (μg/L)	190	37.0 [2.5, 278.3]	107	26.0 [3.7, 146.0]	*83*	31.4 [2.5, 278.3]
Plasma hs-CRP (mg/L)	190	2.2 [0.0, 31.7]	107	0.6 [0.0, 31.7]	*83*	1.3 [0.1, 18.7]**
Blood Pb (μg/L)	191	5.9 [1.78 50.4]	107	5.6 [1.8, 50.4]	84	6.4 [2.40, 28.60]
Urinary cotinine (ng/mL)	190	122.1 [0.6, 2700.0]	*106*	1.5 [0.6, 1500.0]	84	10.3 [0.6, 2700]**
Categorical variables	*n*	% [95% CI]	*n*	% [95% CI]	*n*	% [95% CI]
Age categories						
3–5 years	*35*	18.3 [13.5, 24.4]	*22*	20.6 [14.0, 29.2]	*13*	15.5 [9.1, 24.8]
6–11 years	*77*	40.3 [33.6, 47.4]	*45*	42.1 [33.1, 51.5]	*32*	38.1 [28.5, 48.8]
12–19 years	*79*	41.4 [34.6, 48.5]	*40*	37.4 [28.8, 46.8]	*39*	46.4 [36.4, 57.0]
Girls	*93*	47.1 [40.2, 54.2]	*55*	48.6 [39.3, 58.0]	*38*	45.2 [36.2, 57.0]
BMI categories (*n* = 189)						
Underweight	*2*	1.1 [0.3, 3.8]	*2*	1.9 [0.5, 6.6]	*0*	–
Normal	*59*	31.2 [25.4, 38.1]	*45*	42.1 [33.1, 51.5]**	*14*	17.1 [10.3, 26.8]
Overweight	*52*	27.5 [21.6, 34.3]	*37*	34.6 [26.2, 44.0]*	*15*	18.3 [11.4, 28.0]
Obese	*76*	40.2 [33.5, 47.3]	*23*	21.5 [14.8, 30.2]	*53*	64.6 [53.8, 74.1]**
Parental education						
Primary	*46*	24.1 [18.6, 30.6]	*20*	18.7 [12.4, 27.1]	*26*	31.0 [22.1, 41.5]*
Secondary	*106*	55.5 [48.4, 62.4]	*63*	58.9 [49.0, 68.3]	*43*	51.2 [40.7, 61.6]
College or above	*39*	20.4 [15.3, 26.7]	*24*	22.4 [15.6, 31.2]	*15*	17.9 [11.1, 27.4]
Anemia	*32*	16.8 [12.2, 22.8]	*18*	16.8 [10.9, 25.0]	*14*	16.9 [10.3, 26.3]
3–5 years	*2*	5.9 [1.6, 19.1]	*1*	4.6 [0.1, 21.8]	*1*	8.3 [14.9, 35.4]
6–11 years, girls	*5*	14.7 [6.5, 30.1]	*3*	14.3 [5.0, 34.5]	*2*	15.4 [4.3, 42.2]
6–11 years, boys	*9*	20.9 [11.4, 35.2]	*3*	12.5 [4.3, 31.0]	*6*	31.6 [15.4, 54.0]
12–19 years, girls	*10*	24.4 [13.8, 39.3]	*7*	33.3 [17.2, 54.6]	*3*	15.0 [5.2, 36.0]
12–19 years, boys	*6*	15.8 [7.4, 30]	*4*	21.1 [8.5, 43.0]	*2*	10.5 [2.9, 31.4]
Severity of anemia						
Mild	*20*	10.5 [6.9, 15.7]	*9*	8.4 [4.5, 14.2]	*11*	13.6 [7.6, 22.2]
Moderate	*11*	5.8 [3.3, 10.1]	*9*	8.4 [4.5, 15.2]	*2*	2.4 [0.7, 8.4]
Severe	*1*	0.5 [0.0, 2.9]	*0*	–	*1*	1.2 [0.2, 6.5]
Types of anemia						
IDA	*16*	8.4 [5.3, 13.2]	*10*	9.4 [5.2, 16.4]	*6*	7.2 [3.4, 14.9]
ACI	*12*	6.3 [3.7, 10.7]	*6*	5.6 [2.6, 11.7]	*6*	7.2 [3.4, 14.9]
UA	*4*	2.1 [0.8, 5.3]	*2*	1.9 [0.1, 6.6]	*2*	2.4 [0.7, 8.4]
ID (*n* = 190)	*39*	20.5 [15.4, 26.8]	*22*	20.6 [14.0, 29.2]	*18*	20.5 [13.2, 30.4]
3–5 years	5	14.7 [6.5, 30.1]	*5*	22.7 [10.1, 43.4]	*0*	–
6–11 years, girls	*3*	8.8 [3.1, 23.0]	*1*	4.8 [0.9, 22.7]	*2*	15.4 [4.3, 42.2]
6–11 years, boys	*7*	16.3 [8.1, 30.0]	*1*	4.2 [0.7, 20.2]	*6*	31.6 [15.4, 54.0]
12–19 years, girls	*18*	43.9 [29.9, 59.0]	*11*	52.4 [32.4, 71.7]	*7*	35.0 [18.1, 56.7]
12–19 years, boys	*6*	15.8 [7.4, 30.4]	*4*	21.1 [0.9, 43.3]	*2*	10.5 [29.0, 31.4]
Inflammatory status (hs-CRP > 5 mg/L)	*21*	11.1 [7.3, 16.3]	*9*	8.4 [4.5, 15.2]	*12*	14.5 [8.5, 23.6]
Cigarette smoke exposure (urinary cotinine > 100 ng/mL)	*28*	14.7 [10.4, 20.5]	*9*	8.4 [4.5, 15.2]	*19*	22.6 [15.0, 32.7]**

^a^Arithmetic mean

*GM*, geometric mean; *Min*, minimum value; *Max*, maximum value; *CI*, confidence interval; *BMI*, body mass index; *Hb*, hemoglobin; *SF*, serum ferritin; *hs-CRP*, highly sensitive C-reactive protein; *N*, number of participants; *Pb*, lead; *IDA*, Iron deficiency anemia; *ACI*, anemia of chronic inflammation; *UA*, unknown anemia; *ID*, iron deficiency

*p value < 0.05; **p value < 0.01 for the corresponding *t* test and *χ*^2^ test

To minimize selection bias, a total of 177 participants were randomly selected out of 279 candidates contacted from the lists of potential participants aged 3 to 19 years old and provided by the four community partners. These were recruited according to the underlying population distribution in each of the four communities (3–5 years, 6–11 years, and 12–19 years for both sexes) based on the 2014 Statistics Canada Census (Government of Canada 2014). To reach our recruitment target, 21 additional participants were recruited on a voluntary basis but in accordance with recruitment targets by age and sex groups. Overall, out of the 198 participants recruited, 95% of participants (*n* = 106/111) from the two Anishinabe communities and 82% of participants (*n* = 71/87) from the two Innu communities were selected on a random basis.

For data collection, parents or legal guardians accompanied study participants aged less than 18 years old in all sessions, while older participants completed all sessions independently. After signing an informed consent form and seeking child verbal assent, two qualified nurses administered a short medical questionnaire and collected anthropometric measurements (height and weight), blood samples (by venipuncture), and a spot urine sample. Then, participants and parents/legal guardians were invited to answer an interview-administered questionnaire including education, housing conditions, and household food security information as well as a traditional and market food frequency questionnaire (FFQ). The entire session lasted approximately 1.5 h. To facilitate recruitment, participants were not asked to fast prior to data collection.

### Laboratory analyses

Hb concentration was quantified in situ using a HemoCue Hb 201+. Biological samples were kept frozen at − 20 °C until analyzed. Serum ferritin (SF), transferrin saturation (TS), serum iron (SI), unsaturated iron binding capacity (UIBC), and plasma hs-CRP (high-sensitivity C-reactive protein) were measured at the *Institut Universitaire de Cardiologie et Pneumologie de Québec* using Modular P, Modular E170, and Cobas Integra 800 analyzers. The total iron binding capacity (TIBC) was calculated using the sum of UIBC and SI. Blood Pb was also quantified by inductively coupled plasma mass spectrometry (ICP-MS) at the *Centre de Toxicologie du Québec* (CTQ) of the *Institut national de santé publique du Québec*. Its limit of detection was < 1.04 μg/L.

### Assessment of anemia and ID

**Fig. 2:**
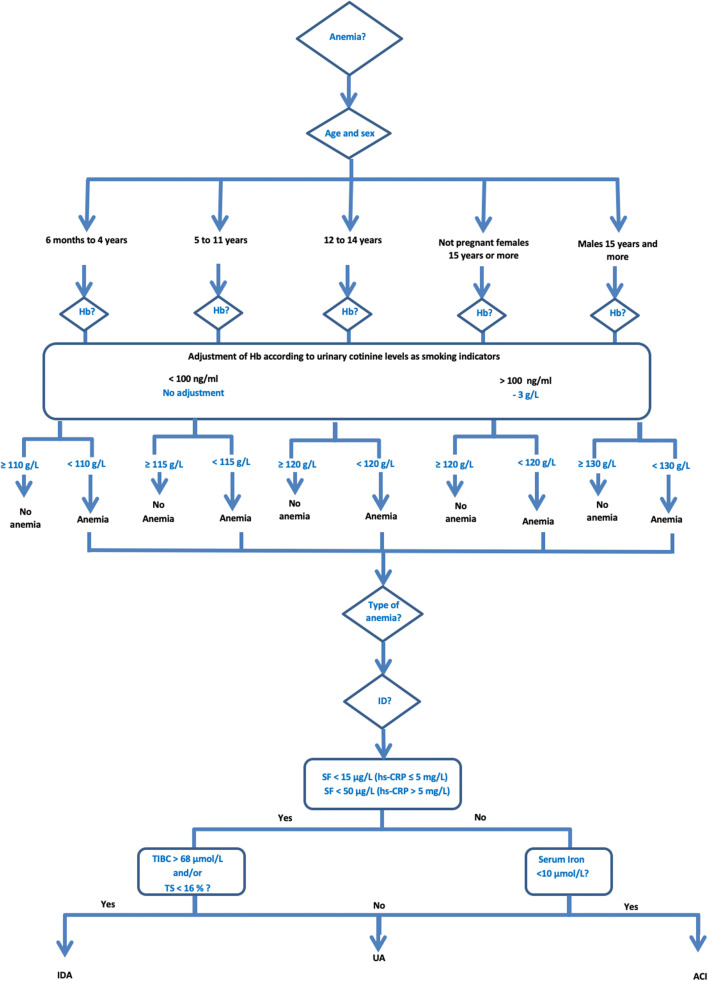
Algorithm for classification of anemia

Anemia was defined as insufficient Hb based on the World Health Organization (WHO) Hb cutoff values specific to each age group and sex (Fig. 2), and further classified into “mild,” “moderate,” or “severe” (WHO 2001). Hb concentrations were adjusted by a factor of − 3 g/L for active cigarette smokers (urinary cotinine > 100 ng/mL) (Nordengberg et al. 1990). No adjustment was made for the altitude given that all study participants were living at sea level (WHO 2001).

ID was assessed based on SF, hs-CRP, and SI levels. Level of SF is a reliable tool to diagnose ID in the absence of inflammation (hs-CRP ≤ 5 mg/L); therefore, SF < 15 μg/L was used to define ID. In the presence of inflammation (hs-CRP > 5 mg/L), which is frequent among children and in First Nations context, a correction is necessary and ID was diagnosed with SF < 50 μg/L (Turgeon O’Brien et al. 2016). Since the maximum level of CRP is reached 48 h following an acute inflammation and decreases gradually to less than 10 mg/L in approximately 10 days, a lower cutoff value of 5 mg/L was used (Turgeon O’Brien et al. 2016).

Participants with anemia were categorized into IDA, ACI, and UA using multiple-indices model as proposed by Plante et al. (2011). When participants with anemia presented with ID and abnormal TIBC and/or TS (> 68 μmol/L and/or < 16%, respectively), they were categorized as IDA (Cash and Sears 1989). Participants with anemia showing low SI (< 10 μmol/L) but without evidence of ID were categorized as ACI as low SI is a consistent feature of chronic inflammation (Cash and Sears 1989); otherwise, they were classified as UA (Plante et al. 2011). Participants diagnosed with anemia on the site of the study were readily referred to health centres and community nutritionists for a follow-up. Moreover, participants’ results for blood metals and nutritional status were returned in person to the parents or legal guardian, and local clinical interventions were undertaken when relevant.

### Statistical analysis

To respect the normality assumptions, a log-transformation was used for Hb, SF, blood and plasma metals, and dietary intake variables. Descriptive analyses were performed (geometric means (GM), 95% confidence intervals (CI), or range). The Student *t* test was used to compare continuous variables, whereas chi-square (*χ*^2^) was used to compare proportions between nations and sex. Dietary variables with extreme values (outliers) were winsorized to the 97.5th percentile. Frequently consumed food (fruits, vegetables, and fruit juice, i.e., natural and powdered juice) and nutrient intakes were used as continuous variables in grams per day (method for nutrient intake calculation is detailed in the Supplementary Materials). Less frequently consumed food variables such as wild fish, birds and game meat, beef meat, pork meat, and chicken were dichotomized (above and below their median value since many participants negated their consumption). Protective and risk factors and confounding variables potentially associated with different outcomes (anemia or ID) are listed in Table S1. Height and weight were used to calculate BMI (kg/m^2^). Since BMI is known to be a less-sensitive single measurement to assess adiposity in children, its *z*-score equivalent to ± 2 SD beyond the means of BMI for age and sex was calculated (Cole et al. 2005).

Structural equation modeling (SEM) was preferred over conventional multiple regression analyses since we were interested in better understanding the contribution of direct and indirect paths of variables associated with the outcomes (Nachtigall et al. 2003). SEM is made of two components: (i) a measurement model to assess co-linearity and create latent variables accordingly; and (ii) a structural model to evaluate the correlations between co-variables. The measurement part of SEM is based on confirmatory factor analysis, where correlated variables (Pearson *r* > 0.40) such as food frequencies or dietary intakes measuring common concepts were identified and combined into single latent variables. To improve models fit, some variables were excluded such as those for infrequently consumed food failing to converge into the latent variables or presenting negligible association with the outcome variables. More details on the complete list of the variables tested and included in the final models are presented in Table S1.

**Fig. 3:**
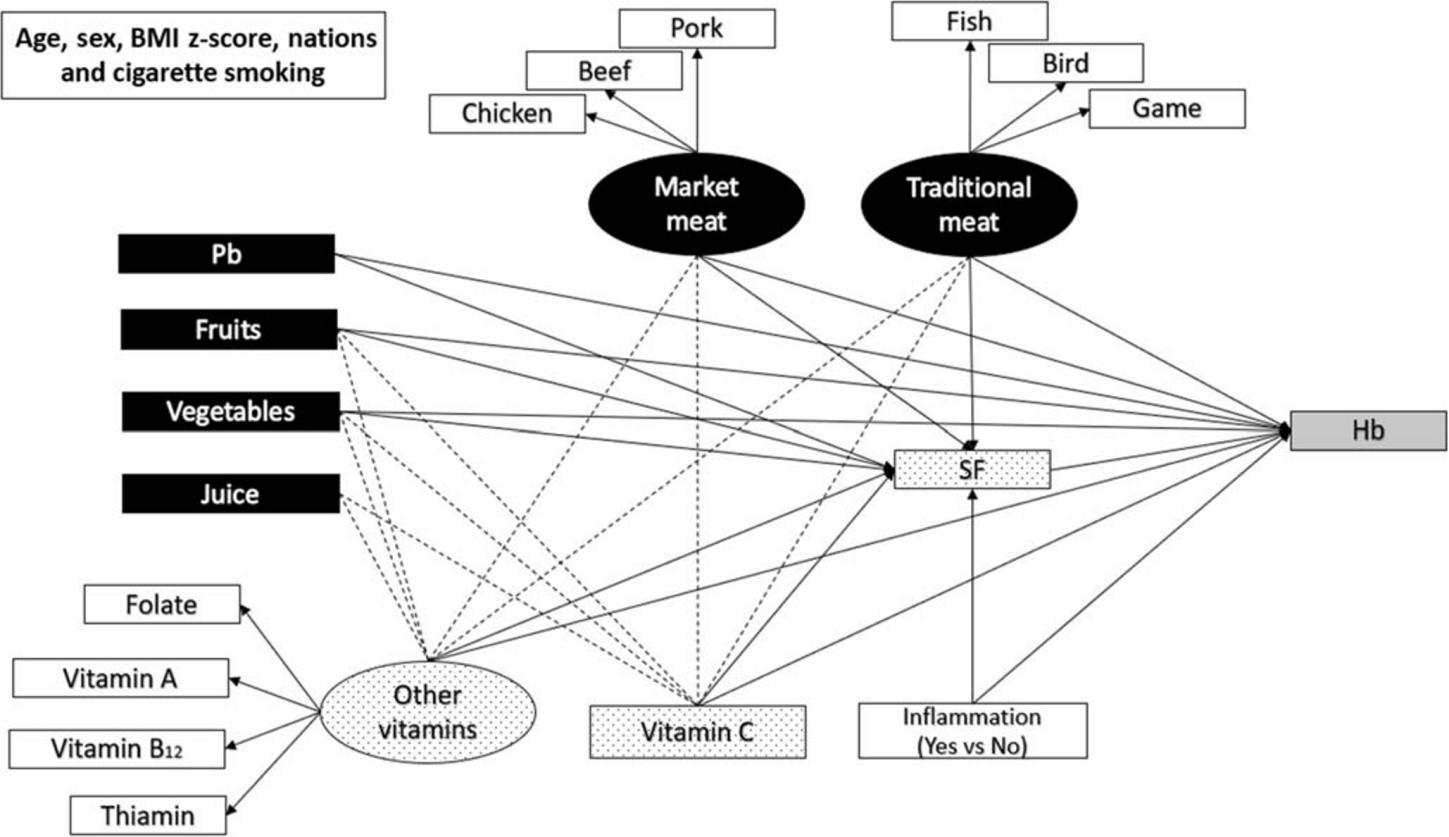
SEM models 1 and 2, showing measured variables (rectangle boxes), latent variables (oval boxes), their items (white boxes), and their direct and indirect effects tested on SF and Hb. Direct effects of the variables on Hb and SF are depicted using uninterrupted lines. Indirect effects are shown using interrupted lines. Gray boxes indicate outcome variables. Black and dotted pattern boxes are predictors and intermediate variables, respectively. No lines were drawn for potentially confounding variables (age, sex, BMI *z*-score, studied nations, and cigarette smoke exposure) since they may affect all associations

The structural component of SEM is depicted in Fig. 3. First, the magnitude of the associations through direct and indirect paths between protectors and risk factors/latent variables and SF (outcome variable) and via intermediate variables (vitamin C intake and other vitamins intakes) was evaluated using regression models. Second, a model evaluating the direct paths between independent variables and Hb (outcome variable) as well as their indirect paths via SF, vitamin C, and other vitamins (intermediate variables) was assessed. The effect of juice consumption was presumed to be exclusively via the vitamin C intake and the other vitamins since processed fruit juices are low in iron and proteins which does not justify a direct path to SF and Hb. Sensitivity analyses testing models stratified by sex and by studied nations were conducted. SEM models fit (provided with data tables in the Supplementary Materials) was assessed based on a *p* value > 0.05 (*χ*^2^ test), root means square error of approximation (RMSEA) less than 0.07, Tucker-Lewis fit index (TLI) greater than 0.95, comparative fit index (CFI) greater than 0.95, and weighted root mean square residual (WRMR) > 0.05 (Hooper et al. 2008). The direct and mediated paths were evaluated using the bootstrap method in which random re-sampling with replacement for 10,000 bootstraps was done to improve estimate accuracy. The two-tailed statistical significance level was fixed at 0.05. The effect of variables was considered significant if *p* value < 0.05 and when the coefficient CI did not include zero. Descriptive statistics were done using SAS version 9.4. SEM analysis was conducted using Mplus software, which by default analyze maximum (*n*) of data using the full information maximum likelihood estimator to account for the few missing values.

## Results

Of the 198 participants recruited, five participants were excluded due to missing blood samples and two participants were excluded due to unrealistic very extreme values in the FFQ.

Anemia and ID prevalences were 16.8% and 20.5% respectively (Table 1) and not different between Anishinabe and Innu participants. More than 20% of 6- to 11-year-old boys and 12- to 19-year-old girls were classified with anemia and almost half (43.9%) of girl participants aged 12 to 19 years old displayed ID. Within both First Nations, IDA was the most frequent type of anemia (*n* = 16/32, 50.0%), and in most cases, anemia was classified as mild or moderate.

Obesity, inflammation, and cigarette smoke exposure were higher among Innu than among Anishinabe participants (Table 1). Moreover, SF levels were lower and ID prevalence was higher among female compared with male participants (Table S2). Blood lead levels were higher among Innu and male participants (Tables 1 and S2) but more than 10 times lower than those reported to impair erythropoiesis.

As shown in Table 2, market meats were on average over twice more consumed than traditional meats. Wild fish and pork were more consumed among Innu participants, while fruits and vegetables were more consumed by Anishinabe participants. For both studied nations, fruits were more consumed than vegetables. Fruit juice consumption (natural and powdered) was higher than water consumption among Innu participants. No significant difference was observed between nations for total market meats, beef, pork, and chicken intakes, nor for vegetables or juice intakes. With the exception of fruits and pork meat, which were more consumed by girls, other food intakes were not different between sexes (Table S3). More than 90% of participants had adequate intakes of iron, vitamin A, vitamin B_12_, vitamin C, folate, and thiamin (Table 2), and no differences between nations or sexes were found (Tables 2 and S3).

**Table 2:** Daily food item consumption and dietary intakes and proportions of participants with adequate dietary intakes for all study participants and by nations

**Food and dietary intake variables (g/day)**	**All participants (*n*** **= 191)**	**Anishinabe (*n*** **= 107)**	**Innu (*n*** **= 84)**
	**GM [95% CI]**	**GM [95% CI]**	**GM [95% CI]**
Traditional meats	28.2 [23.5, 33.8]	25.2 [19.7, 32.3]	33.0 [25.3, 43.0]
Wild fish	20.6 [16.3, 26.1]	15.0 [10.5, 21.5]	26.8 [19.9, 36.3]*
Game	14.5 [11.9, 17.7]	15.4 [12.0, 19.8]	12.5 [9.3, 16.8]
Wild birds	13.4 [10.9, 16.5]	15.0 [11.0, 20.5]	12.0 [9.0, 16.0]
Market meats	67.6 [60.5, 75.6]	68.7 [58.6, 80.6]	66.2 [56.2, 77.8]
Beef	22.2 [19.5, 25.4]	23.0 [19.0, 27.8]	21.3 [17.8, 25.6]
Pork	14.5 [12.7, 16.5]	12.3 [10.3, 14.7	17.8 [14.7, 21.5]**
Chicken	31.4 [27.7, 35.7]	34.9 [29.2, 41.7]	27.9 [23.3, 33.4]
Fruits	184.7 [162.8, 209.5]	205.4 [176.9, 238.5]*	161.2 [130.1, 199.8]
Vegetables	64.1 [54.8, 75.0]	74.6 [61.0, 91.2]*	53.1 [41.5, 68.1]
Juice (mL/day)	437.8 [385.5, 497.3]	457.2 [391.5, 533.8]	413.2 [332.5, 513.4]
Water (mL/day)	398.6 [347.3, 457.4.]	461.0 [389.7, 545.2]*	327.1 [260.3, 411.0]
**Micronutrients intake (mg/day)**	**GM [95% CI]** **% Adequate intake**^**a**^	**GM [95% CI]** **% Adequate intake**^**a**^	**GM [95% CI]** **% Adequate intake**^**a**^
Vitamin A	851.1 [768.8, 942.2] 94.9	851.1 [768.8942.2] 96.4	772.3 [695.9, 857.1] 92.90
Vitamin B_12_	7.9 [7.3, 8.5] 100.0	7.8 [7.0, 8.6] 100.0	8.1 [7.1, 9.1] 100.0
Folate	543.4 [511.3, 577.7] 96.4	522.2 [482.5, 565.3] 98.2	572.4 [519.9, 630.3] 94.0
Thiamin	2.5 [2.4, 2.7] 100.0	2.5 [2.4, 2.7] 100.0	2.6 [2.3, 2.8] 100.0
Vitamin C	214.3 [195.7, 234.8] 96.4	226.7 [203.9, 252.0] 97.3	199.2 [169.9, 233.6] 95.2
Iron	17.4 [16.3, 18.5] 99.5	17.4 [16.0, 18.9] 100.0	17.3 [15.7, 19.1] 98.8

^a^Adequate intake was estimated based on Health Canada reference values on age and sex cutoffs of average intake and adequate intakes based on recommended daily allowances (Health Canada, 2006)

*GM*, geometric mean; *CI*, confidence interval

*p value < 0.05, **p value < 0.01 for the corresponding *t* test

**Fig. 4:**
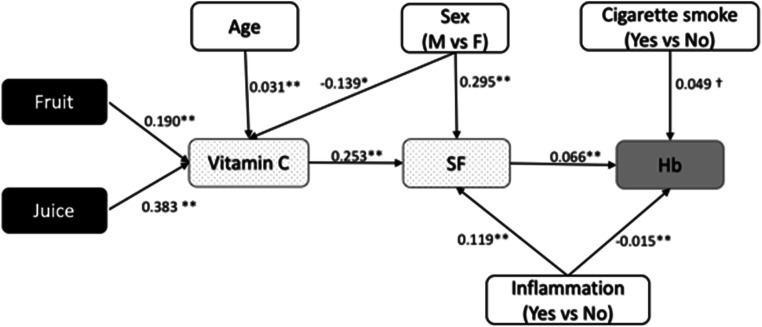
Significant direct associations (not standardized coefficients) between dietary and non-dietary determinants of SF and Hb (models 1 and 2) for all study participants (*n* = 191). Significance level: ^†^*p* value < 0.10, *p value < 0.05, **p value < 0.01

Results of models 1 and 2 evaluating the associations between potential protective and risk factors and SF and Hb levels are shown in Fig. 4. All significant direct and indirect variable coefficients are detailed in Table S4. Model 1 results of SEM analysis showed that male sex, inflammation, and vitamin C intake were associated with higher SF. Moreover, vitamin C intake was found to mediate the positive association between fruit and juice consumption and SF. The direct association between fruit consumption and SF was not statistically significant (data not shown; coefficient for the direct path [95% CI], − 0.009 [− 0.296, 0.291]). All other protective and risk factor variables, including food variables rich in iron and/or proteins, were not directly or indirectly related to SF.

For model 2, cigarette smoking and higher levels of SF were in turn associated with higher Hb concentrations, whereas inflammation was associated with lower Hb levels. As for SF, fruit and juice consumptions were positively associated with Hb, mediated via vitamin C and SF. No significant direct or indirect associations were identified between Hb and other variables, including blood Pb levels.

Analogous consistent results with some differences were noticed when stratifying two models by studied nations and by sex (Supplementary Materials Tables S5 and S6). Indeed, the associations mediated by vitamin C were significant only in girl participants (Table S5). Conversely, among male participants, inflammation status was the main variable associated with SF and Hb, as was traditional meat consumption with higher Hb via SF. Moreover, girls’ age was inversely associated with SF, while the contrary was observed in boys. For models stratified by nations (Table S6), similar trends for fruit and juice consumption via vitamin C intake, sex, and inflammation were observed as in the unstratified models, although in some cases the associations did not reach statistical significance. Cigarette smoke exposure was positively associated with Hb among Innu participants where its exposure was significantly higher. As in the sex-stratified model, traditional meat consumption was associated with higher SF (but only via vitamin C) and with higher Hb via vitamin C and SF in Anishinabe participants.

## Discussion

In spite of adequate iron and vitamin intake among most JES!-YEH! participants, prevalence of anemia and ID among Anishinabe and Innu participants in 2015 was elevated (16.8% and 20.5% respectively), and much higher than in the Canadian general youth population, which was only about 3% in 2009–2011 (Cooper et al. 2012). These high prevalences are comparable to those reported for other Indigenous children in North America, emphasizing the important health disparities between First Nations and other children in Canada and the United States (Jamieson and Kuhnlein 2008). The WHO considers anemia as a public health problem whenever its prevalence exceeds 5%. Accordingly and in spite of using capillary blood to determine Hb using HemoCue analyzer, which is known to overestimate the Hb concentrations compared with the complete blood count method (Hinnouho et al. 2018), the anemia prevalence in JES!-YEH! was more than three times this limit. Interestingly, higher traditional meat, fruit, and fruit juice consumption via increased vitamin C intake and lower inflammation status were the main determinants associated with lower ID and anemia.

In our study, the vast majority of participants with anemia were in the mild or moderate severity categories (97.0% of participants with anemia). Nevertheless, considering the systemic impacts of mild-to-moderate hemoglobin deficiency on children’s and adolescents’ development, addressing underlying factors associated with chronic anemia is important for youth success in school and later in life (Turkoski 2003; Teni et al. 2017). Moreover, low iron status, with or without anemia, which was found in 20.5% of our study participants, is also known to impair optimal youth development, as it also contributes to lower cognitive functions, thermoregulation, and immunity in children and adolescents (WHO 2001).

The most prevalent type of anemia in our study was IDA. Indeed, as iron is an absolute requirement for Hb synthesis, IDA is known as a major contributor to childhood anemia burden globally (WHO 2001). The second most prevalent type of anemia was ACI, whereas the prevalence of anemia due to other causes (UA) was relatively low, which is in agreement with the observation that vitamin intakes were adequate in most of the study participants. Other possible causes of anemia such as parasites, hemorrhages, hemolytic anemia, and hemoglobinopathies are possible but were not assessed in the present study.

In the SEM analysis, SF was consistently positively associated with Hb, even after stratification by sex and nations. SF increased with age among boys, but decreased with puberty among girls due to the increasing demands for iron related to blood loss in adolescent girls (WHO 2001). SF was also positively associated with inflammation since SF is an acute phase reactant whose synthesis and release increase during acute inflammation (WHO 2001). Conversely, inflammation was inversely associated with Hb. Indeed, excessive release of inflammatory cytokines is known to impair erythropoietin synthesis and the production of Hb (Jamieson et al. 2012). It is also known to provoke a functional state of ID, by causing a sequestration of iron in its stores, preventing its transport and utilization, and in turn the integration of iron into Hb (Hurrell 2012). In the present study, we only used hs-CRP to assess inflammation status. Considering that this biomarker is known to be less sensitive in the case of chronic inflammation (Gruys et al. 2005), which was possibly quite high in our study population considering the very high prevalence of overweight and obesity, the prevalence of inflammation in our study population was likely underestimated. Still, up to 11% of our study participants presented an inflammatory status, perhaps due to high prevalence of *H. pylori* infection, or chronic ear and upper respiratory tract infections, as observed in another study among preschool Inuit children (Pacey et al. 2011).

In the SEM analysis, our proxy for iron status (SF) was positively associated with vitamin C intake and, interestingly, vitamin C acted as a mediator of the positive association between fruit and juice consumption and SF, and ultimately Hb. Vitamin C plays an important role in iron metabolism (Lane and Richardson 2014). For instance, vitamin C transforms non-heme iron from a ferric state to a more water-soluble and more absorbable ferrous form (WHO 2001; Lane and Richardson 2014). Consequently, vitamin C intake enhances non-heme iron absorption in the small intestine (WHO 2001). Based on the FFQ, most of the participants had sufficient dietary intakes of food containing vitamin C and iron. Thus, the positive associations between vitamin C and SF, and then with Hb, possibly reflect a higher vitamin C requirement due to the high inflammation status in this context (Gruys et al. 2005). Indeed, as reported by Engle-Stone and colleagues (Engle-Stone et al. 2017) in a recent review among preschool children in a high inflammation context, higher rates of ID and IDA were common and primarily related to iron malabsorption issues (Engle-Stone et al. 2017). It is of note that for vitamin C to increase non-heme iron absorption in food, vitamin C-containing food consumption must be concurrent or shortly after food rich in non-heme iron (WHO 2001; Fishman et al. 1999). Although this information was not captured by FFQ in the present study, we can speculate that greater juice and fruit consumers would likely consume more juice and fruits at mealtimes. Juice was indeed largely consumed by the study participants, and even consumed more often than water.

Despite our small sample size, we found very interesting sex differences. The association mediated by vitamin C on SF and Hb was mainly observed in girls who showed significantly higher intake of vitamin C compared with boys. Conversely, among boys, inflammation was associated with lower Hb, and traditional meat intake was associated with higher SF and Hb concentrations. Comparable results were reported in adult Inuit men and women, for whom anemia was mainly iron-dependent in premenopausal women but inflammatory-related in men (Jamieson et al. 2016). Inflammation is known to antagonize the absorption of iron mediated by vitamin C as it enhances hepcidin release and down regulates feroportin1, an iron transporter in the small intestine (Jamieson et al. 2012; Sears 1992). Traditional meats are high in bioavailable iron, proteins, and vitamin C, all associated with positive iron intake and absorption (Jamieson et al. 2012). Interestingly, traditional meat consumption and inflammation status were not different between boy and girl participants, and this highlights the importance of further investigating sex-based differences in iron metabolism concomitantly with other variables such as high inflammation as well as puberty and menstruation in Indigenous youth context.

Aside from the inherent limitations of cross-sectional studies to establish causality, in the present study, the recruitment of few voluntary participants limits the generalization of our results beyond the studied participants. Moreover, the FFQ used in this study presents important limitations as principal care giver responded for younger children without fully knowing what their children eat all day (like snacks provided in school, but the children come home for lunch), particularly in cases where parents were separated. The FFQ may also have led to an underestimation of food and nutrient intakes and further studies should consider using repeated 24-h recalls (Shim et al. 2014), although this represents a significant challenge in remote Indigenous study context when time is limited. Next, in order to classify ACI with no evidence of ID, we used as criterion SI < 10 μmol/L. As a result of non-fasting status prior to data collection, misclassification of some participants with UA as ACI is expected due to higher estimation of SI. Finally, further studies should consider better assessing inflammation status using multiple biomarkers simultaneously (i.e., hs-CRP and alpha1-acid glycoprotein) and documenting sources of chronic inflammation, infections, and blood loss, such as *H. pylori* active infection and menstruation, as well as properly estimating the type of iron in the diet and the timing of vitamin C intake in respect to meals.

## Conclusion

This pilot project highlights an elevated prevalence of anemia and ID in particularly vulnerable Indigenous subpopulations in Canada. Our findings suggest that intervention fostering healthier food environments and traditional meat consumption as well as higher consumption of foods naturally rich in vitamin C, which is known to enhance iron absorption, and fighting underlying causes of inflammation could decrease ID and anemia. Indeed, these findings raise the importance of better documenting this public health problem among First Nations youth across the country and call for preventive actions at multiple scales.

## Electronic supplementary material

ESM 1 (DOCX 66.2 kb)
