# Supplementary material for: Anemia, iron status, and associated protective and risk factors among children and adolescents aged 3 to 19 years old from four First Nations communities in Quebec
Source: Can J Public Health. 2020 Mar 13;111(5):682–93. doi: 10.17269/s41997-020-00304-7 (PMC7501325; doi:10.17269/s41997-020-00304-7)
Supplement: Supplementary file 1 — (DOCX 66.2 kb) [file 41997_2020_304_MOESM1_ESM.docx]

**Anemia, iron status and associated protective and risk factors among children and adolescents aged 3 to 19 years old from four First Nations communities in Quebec**

**Emad Tahir^1,2^,** Pierre Ayotte^1,2,3^, Matthew Little^1,2^, Richard E. Bélanger^1, 4^, Michel Lucas^1, 2^, Donna Mergler^5^, Elhadji A. Laouan Sidi^2^, Community of Winneway - Long Point First Nation, Community of Lac Simon, CSSS Tshukuminu Kanani of Nutashkuan, Community of Unamen Shipu, Nancy Gros-Louis McHugh^6^, Mélanie Lemire^1,2, *^

^1^ Axe santé des populations et pratiques optimales en santé, Centre de recherche du CHU de Québec - Université Laval, QC, Canada;

^2^Département de médecine sociale et préventive, Université Laval, Québec, QC, Canada ;

^3^Institut national de santé publique du Québec, Québec, QC, Canada ;

^4^Département de pédiatrie, Université Laval, Québec, QC, Canada ;

^5^CINBIOSE, Université du Québec à Montréal, Montréal, QC, Canada ;

^6^ First Nations of Québec and Labrador Health and Social Services Commission, Wendake, QC, Canada

* Corresponding author: Centre de Recherche du CHU de Québec, Hôpital du Saint-Sacrement, 1050 chemin Sainte-Foy, Québec, QC G1S 4L8, Canada. Email address: [melanie.lemire@crchudequebec.ulaval.ca](mailto:melanie.lemire@crchudequebec.ulaval.ca)

## Food consumption and dietary intakes data

The FFQ was used to assess local traditional food consumption by seasons over the last year, and market food and beverage consumption over the past three months (24). Questions were developed based on previous First Nations and Inuit studies (Nieboer et al., 2013; Rochette & Blanchet, 2004), and piloted with community partners and volunteers from Anishinaabe and Innu nations. Posters with images and names in English, French, Anishinaabe or Innu languages of all traditional foods were used to provide a visual support.

To minimize the recall bias related to portion sizes estimation, adults’ standard portion sizes in grams (g) were estimated for each food item of the FFQ (281 items) based on portion size assessed in the *2005–2009 Nituuchischaayihtitaau Aschii* Multi-community Environment-and-Health Study in *Iiyiyiu Aschii* (Cree) (26) and the 2004 *Qanuippitaa* Inuit Health Survey (25). When a food item was available in both studies, the most conservative portion (i.e. the lesser of the two) was used. When a portion of a specific meat part of an animal was not available, portion size from another equivalent part in the same animal was used (i.e. heart to liver); if the information was not available (i.e. brain, bone marrow or intestines), portions of similar parts from another animal was used. Portions for some traditional foods were not available from either study (i.e. seafood like lobster, snow crab or shrimps), and in such cases, these were assumed to be equivalent to other similar foods (i.e. shrimps to scallops). Portions of beverages were used directly as they were surveyed directly in the FFQ. Age-adjusted portions were computed from adults’ standard portions or portions provided by the FFQ, and food frequencies were divided by their respective age-adjusted portions to assess each food item intake in grams per day.

The average nutrient (iron, vitamins etc.) daily intakes (in mg/day) were calculated based on food intakes (g/day). For traditional foods, food intake data for the past season (spring for Anishinaabe, and average of summer and fall for Innu communities) were used whereas for market foods, data for the past three months were used. The Canadian Nutrient File (CNF) was used to assess nutrient contents (per 100 g) of each traditional and market food. The CANDAT software (Godin, London, Ontario) was used to assign nutrients to specific food. If a food item was not available on the CNF, nutrient contents were derived from the CINE database for traditional food (27), other international databases such as INFOODS or an estimate of a similar food. To identify participants with adequate or inadequate intakes, age-specific cut-offs of estimated average intake and adequate intakes based on Health Canada reference values were used (28).

Table S1: Determinants of serum ferritin (SF) and hemoglobin (Hb) tested in the structural equation model and variables included in the final models

| **Inclusion criteria** | **Sociodemographic and biological factors*^a^*** | **Commonly consumed food and dietary variable** | **Iron-absorption inhibitors*^a^*** | **Iron-absorption enhancers** |
| --- | --- | --- | --- | --- |
| **Tested variables** | -Age  -Sex  -BMI z-score*^b^*  -Food security*^c^*  -Overcrowding  -Education  -Self perceived health  - Cigarette smoke exposure  -Inflammation  -Lead exposure (blood Pb)  -Household*^d^*  -Community^d^  -Nations*^d^*  -Interviewer*^d^* | - Market (beef, chicken, pork, croquette, jerky meat, hotdog, sausage, canned fish and transformed meat)  - Traditional meat (game, birds, wild fish, and seafood)  - Fruits  - Vegetables  -Juice  -Vitamin B_12_ intake  -Folate intake  -Vitamin D intake  -Thiamin intake  - Wild berries  - Beans  - Nuts | -Eggs  -Tea  -Coffee  -Cereals  -Dairy products  -Mn intake  -Zn intake  -Ca intake | -Iron intake  -Vitamin C intake  -Vitamin A intake  -Oral supplements |
| **Variable selected for final models^1^** | -Age  -Sex  -BMI z-score  - Cigarette smoke exposure  -Inflammation  -blood Pb  -Nations*^d^* | - Market meat (beef, chicken and pork meats)  - Traditional meat (game, birds, wild fish)  Fruits  -Vegetables  -Juice  - Vitamin B_12_ intake  -Folate intake  -Thiamin intake |  | -Vitamin C intake  -Vitamin A intake |

^1^ Main reasons for exclusions were infrequent consumed for food items, failure to converge into latent variables and variables presenting negligible associations with the outcomes. *^a^*Jamieson & Kuhnlein (2008), Meltzer (2010), and World Health Organization, UNICEF (2001), *^b^*Cole et al (2005); *^c^*US Department of Health and Human Services (2007); *^d^*Potential confounders.

Table S2: Characteristics of the study participants by sex (n=191)

|  | **Variables** | | **Girls (n=90)** | | | **Boys (n=101)** | |
| --- | --- | --- | --- | --- | --- | --- | --- |
|  |  |  | **N** | **GM [Min-Max]** | **N** | | **GM [Min-Max]** |
| **Continuous variables** | | |  |  |  | |  |
|  | **Age in years *^a^*** | |  | 9.8 [3, 19] |  | | 10.6 [3, 19] |
|  | **BMI** | |  | 23.0 [14.3, 46.4] | **99** | | 23.1[15.6, 64.7] |
|  | **Hb (g/L)** | |  | 123.1 [88.0, 160.0] |  | | 126.5 [60.0, 175.0] |
|  | **SF (μg/L)** | | **89** | 24.6 [2.5, 278.3] |  | | 31.7 [3.3, 184.3]* |
|  | **Plasma hs-CRP (mg/L)** | | **89** | 0.9 [0.0, 18.7] |  | | 0.9 [0.0, 31.7] |
|  | **Blood Pb (µg/L)** | |  | 5.1 [1.8, 50.4] |  | | 6.8 [2.4, 28.6]* |
|  | **Urinary cotinine (ng/mL)** | |  | 4.0 [0.6, 2700.0] | **100** | | 3.2 [0.6, 1500.0] |
| **Categorical variables** | | | **N** | **% [95%CI]** | **N** | | **% [95%CI]** |
|  | **Age categories** | |  |  |  | |  |
|  |  | 3 - 5y | **15** | 16.7 [10.4, 25.7] | **20** | | 19.8 [13.2, 28.6] |
|  |  | 6 - 11y | **34** | 37.9 [28.5, 48.1] | **43** | | 42.6 [33.4, 52.3] |
|  |  | 12 - 19y | **41** | 45.6 [35.7, 56.4] | **38** | | 37.6 [28.8, 47.4] |
|  | **Anishinaabe** | | **38** | 42.2 [32.5, 52.5] | **46** | | 45.5 [36.2, 55.2] |
|  | **BMI Categories** | |  |  |  | |  |
|  |  | Underweight | **0** | - | **2** | | 2.2 [0.5, 7.1] |
|  |  | Normal | **31** | 34.4 [25.5, 44.7] | **28** | | 28.3 [20.3, 37.8] |
|  |  | Overweight | **27** | 30.0 [21.5, 40.1] | **25** | | 25.3 [17.7, 34.6] |
|  |  | Obese | **32** | 35.6 [26.4, 45.9] | **44** | | 44.4 [35.1, 54.3] |
|  | **Parental education** | |  |  |  | |  |
|  |  | Primary | **20** | 22.2 [14.9, 31.9] | **26** | | 25.7 [18.2, 35.1] |
|  |  | Secondary | **49** | 54.4 [44.2, 64.3] | **57** | | 56.4 [46.7, 66.7] |
|  |  | College or above | **21** | 23.3 [15.8, 33.1] | **18** | | 17.8 [11.6, 26.4] |
|  | **Anemia** | | **17** | 19.1 [12.3, 28.5] | **15** | | 14.9 [9.2, 23.1] |
|  | **Severity of anemia** | |  |  |  | |  |
|  |  | Mild | **12** | 13.5 [7.9, 22.1] | **8** | | 7.9 [4.1, 14.9] |
|  |  | Moderate | **5** | 5.6 [2.4, 12.5] | **6** | | 5.9 [2.8, 12.4] |
|  |  | Severe | **0** | ̶ | **1** | | 1.0 [0.0, 5.4] |
| **Types of anemia** | | |  |  |  | |  |
|  | IDA | | **9** | 10.1 [5.4, 18.1] | **7** | | 6.9 [3.4, 13.6] |
|  | ACI | | **6** | 6.7 [3.1, 13.9] | **6** | | 5.9 [2.8, 12.4] |
|  | UA | | **2** | 2.3 [0.6, 7.8] | **2** | | 2.0 [0.5, 6.9] |
|  | **ID** | | **24** | 27.0 [18.8, 37.0]* | **15** | | 14.9 [9.2, 23.1] |
|  | **Inflammatory status (hs-CRP > 5 mg/L)** | | **9** | 10.1 [5.4, 18.1] | **12** | | 11.9 [6.9, 19.6] |
|  | **Cigarette smoke exposure (urinary cotinine >100 ng/mL)** | | **16** | 17.8 [11.3, 26.9] | **12** | | 12 [7.0, 19.8] |

*^a^* arithmetic mean

GM = geometric means; Min = minimum value; Max= maximum value; CI = confidence interval; BMI = Body mass index; Hb = hemoglobin; SF = Serum ferritin; hs-CRP = highly sensitive C reactive protein,

*p*-value < 0.05 the corresponding *t*-test and *X^2^* test

Table S3: Daily food items consumption and dietary intakes and proportions of participants with adequate dietary intakes by sex (n=191)

|  | **Food and dietary intake variables (g/day)** | | **Girls (n=90)** | **Boys (n=101)** |
| --- | --- | --- | --- | --- |
|  |  |  | **GM [95%CI]** | **GM [95%CI]** |
|  | **Traditional meats** | | 30.9 [24.4, 39.0] | 26.2 [19.6, 35.0] |
|  |  | Wild fish | 25.6 [18.5, 35.5] | 17.4 [12.5, 24.3] |
|  |  | Game | 15.2 [12.1, 19.2] | 13.9 [10.0, 19.3]) |
|  |  | Wild birds | 13.9 [10.2, 18.9] | 13.0 [9.6, 17.4] |
|  | **Market meats** | | 74.6 [64.0, 86.9] | 61.8 [52.4, 73.0] |
|  |  | Beef | 23.5 [19.4, 28.5] | 21.1 [17.6, 25.4] |
|  |  | Pork | 16.7 [14.3, 19.6]* | 12.8 [10.4, 25.7] |
|  |  | Chicken | 34.7 [29.5, 41.0] | 28.6 [23.7, 34.7] |
|  | **Fruits** | | 225.9 [194.0, 263.2]** | 154.3 [127.4, 186.8] |
|  | **Vegetables** | | 78.0 [64.2, 94.9]* | 53.8 [42.3, 68.3] |
|  | **Juice (ml/day)** | | 478.0 [402.1, 568.4] | 405.0 [336.1, 488.0] |
|  | **Micronutrients intake** (mg/day) | | **GM [95%CI]**  **%adequate intake*^a^*** | **GM [95%CI]**  **%adequate intake*^a^*** |
|  |  | Vitamin A | 849.1 [768.5,938.2]  87.8 | 786.6 [707.3, 874.9]  100.0 |
|  |  | Vitamin B_12_ | 8.1 [7.3, 9.0]  100.0 | 7.7 [6.8, 8.7]  100.0 |
|  |  | Folate | 559.5 [512.1, 611.2]  98.9 | 534.7 [489.2, 584.3]  98.2 |
|  |  | Thiamin | 2.5 [2.4, 2.7]  98.9 | 2.5 [2.3, 2.7]  100.0 |
|  |  | Vitamin C | 236.6 [209.9, 266.6]  98.9 | 194.6 [169.5, 223.4]  100.0) |
|  | Iron | | 17.9 [16.4, 19.5]  98.9 | 17.0 [15.5, 18.7]  100.0 |

*^a^*Adequate intake was estimated based on Health Canada reference values on age and sex cut-offs of average intake and adequate intakes Health Canada (2006)

GM = geometric means; CI = confidence interval;

*: *p*-value < 0.05, **: *p*-value < 0.01 the corresponding *t*-test

Table S4: Significant direct and indirect associations between sociodemographic, dietary and physiological determinants of serum ferritin (SF) and hemoglobin (Hb) for all study participants (n=191)^a^

| **Predictors** | **Intermediate variables** | **SF (μg/L)** | **Hb (g/L)** |
| --- | --- | --- | --- |
|  |  | **Coefficient [95% CI]** | **Coefficient [95% CI]** |
| Sex (M vs F) | – | 0.295 [0.093, 0.502]** | 0.021 [-0.013, 0.052] |
| SF (μg/L) | – | – | 0.066 [0.040, 0.096]** |
| Inflammation (Yes vs No) | – | 0.119 [0.049, 0.190]** | -0.015[-0.025, -0.005]** |
| Inflammation (Yes vs No) | SF (μg/L) | – | 0.008 [0.004, 0.015]** |
| Cigarette smoke (Yes vs No) | – | -0.180 [-0.554, 0.140] | 0.049 [-0.008, 0.117]† |
| Vitamin C intake (mg/day) | – | 0.253 [0.064, 0.431]** | – |
| Fruit (g/day) | Vitamin C (mg/day) | 0.090 [0.027, 0.161]** | – |
| Fruit (g/day) | Vitamin C (mg/day) and SF (μg/L) | – | 0.004[0.001, 0.010]** |
| Juice (ml/day) | Vitamin C (mg/day) | 0.237 [0.060, 0.411]** | – |
| Juice (ml/day) | Vitamin C (mg/day) and SF (μg/L) | – | 0.008 [0.003, 0.017]** |

Significance level †: *p*-value < 0.10; *: *p*-value < 0.05, **: *p*-value < 0.01

*^a^*Model fit for SF (*χ2* = 146.4, *p* = 0.0001, RMSEA = 0.06 [90%CI; 0.04-0.08], *p* = 0.10; CFI = 0.93; TLI = 0.91; WRMR = 1.11, *R^2^* =19.4%)

*^a^*Model fit for Hb (*χ2* = 259.38, *p <*0.0001, RMSEA = 0.06 [90%CI; 0.05-0.07], *p* = 0.08; CFI = 0.91; TLI = 0.88, WRMR = 1.28, *R^2^* =31.7%)

Table S5: Direct and indirect associations between sociodemographic, dietary and physiological determinants of ferritin (SF) and hemoglobin (Hb) stratified by sex (n=191)^a^

| **Predictors** | **Intermediate**  **variables** | **Girls (n= 90)** | | **Boys (n=101)** | |
| --- | --- | --- | --- | --- | --- |
|  |  | **SF (μg/L)** | **Hb (g/L)** | **SF (μg/L)** | **Hb (g/L)** |
|  |  | **Coefficient [CI 95%]** | **Coefficient [CI 95%]** | **Coefficient [CI 95%]** | **Coefficient [CI 95%]** |
| Age (y) | – | -0.048 [-0.083, -0.010]* | 0.003 [-0.003, 0.008] | 0.039[0.005, 0.071]* | 0.008 [-0.002, 0.015]† |
| SF (μg/L) | – | – | 0.047 [0.004, 0.084]* | – | 0.079 [0.027, 0.245]** |
| Inflammation (Yes vs No) | – | 0.049 [-0.056, 0.147] | -0.016 [-0.029, -0.004]** | 0.150 [0.059, 0.250]** | -0.018 [-0.042, -0.002]* |
| Inflammation (Yes vs No) | SF (μg/L) | – | 0.002 [-0.003, 0.009] | – | 0.012 [0.002, 0.036]** |
| Vitamin C intake (mg/day) | – | 0.493 [0.175, 0.752]** | – | 0.062 [-0.173, 0.257] | – |
| Cigarette smoke (Yes vs No) | – | -0.205 [-0.657, 0.251]† | 0.023 [-0.029, 0.081]† | -0.034 [-0.564, 0.539] | 0.086 [-0.003, 0.221]† |
| Fruit (g/day) | Vitamin C (mg/day) | 0.073 [-0.026, 0.191]† | – | 0.012 [-0.037, 0.063] | – |
| Fruit (g/day) | Vitamin C (mg/day) & SF (μg/L) |  | 0.004 [-0.002, 0.016]† | – | 0.001 [-0.002, 0.011] |
| Juice (ml/day) | Vitamin C (mg/day) | 0.161 [0.053, 0.265]** | – | 0.027 [-0.081, 0.117] |  |
| Juice (ml/day) | Vitamin C (mg/day) & SF (μg/L) | – | 0.010 [0.001, 0.024]* | – | 0.003 [-0.005, 0.018] |
| Traditional food (g/day) | SF (μg/L) | – | -0.004 [-0.043, 0.007] | – | 0.014 [0.000, 0.065]* |

Significance level †: *p*-value < 0.10; *: *p*-value < 0.05, **: *p*-value < 0.01

*^a^*Model fit for SF (*χ2* = 231.48, *p* = 0.0003, RMSEA = 0.07 [90%CI; 0.05-0.09], *p* = 0.09; CFI = 0.91; TLI = 0.89; WRMR = 1.1246)

*^a^*Model fit for Hb (*χ2* = 418.33, *p <*0.0001, RMSEA = 0.07 [90%CI; 0.05-0.08], *p* = 0.95; CFI = 0.91; TLI = 0.85, WRMR = 1.37)

SF = Serum ferritin; Hb = Hemoglobin; CI = Confidence interval;

Table S6: Significant direct and indirect associations between sociodemographic, dietary and physiological determinants of serum ferritin (SF) and hemoglobin (Hb) for participants stratified by nations (N=191)^a^

| Predictors | Intermediate variables | Anishinaabe (n=107) | | Innu (n=84) | |
| --- | --- | --- | --- | --- | --- |
|  |  | **SF (μg/L)** | **Hb (g/L)** | **SF (μg/L)** | **Hb (g/L)** |
|  |  | **Coefficient [CI 95%]** | **Coefficient [CI 95%]** | **Coefficient [CI 95%]** | **Coefficient [CI 95%]** |
| Sex (M vs F) | – | 0.213 [-0.026, 0.468]† | 0.31 [-0.010, 0.073] | 0.397 [0.062, 0.751]* | 0.007 [-0.071, 0.056] |
| SF (μg/L) | – | – | 0.088 [0.033, 0.133]** | – | 0.051 [0.010, 0.203]** |
| Cigarette smoke (Yes vs No) | – | - 0.294 [-0.748, 0.222] | 0.032 [-0.044, 0.093] | -0.076 [-0.712, 0.504] | 0.084 [0.005, 0.242]* |
| Inflammation (Yes vs no) | – | 0.098 [0.029, 0.170]** | -0.018 [-0.031, -0.006]** | 0.183 [-0.018, 0.376]† | -0.008 [-0.046, 0.008] |
| Inflammation (Yes vs no) | SF (μg/L) | – | 0.009 [0.001, 0.020]* | – | 0.009 [0.000, 0.054]* |
| Vitamin C intake (mg/day) | – | 0.340 [0.008, 0.613]* | – | 0.290 [-0.066, 0.530]† | – |
| Fruit (g/day) | Vitamin C (mg/day) | 0.067 [0.005, 0.188]* | – | 0.049 [0.009, 0.123]* | – |
| Fruit (g/day) | Vitamin C (mg/day) and SF (μg/L) | – | 0.004 [-0.016, 0.015] | – | 0.004 [0.001, 0.017]** |
| Juice (ml/day) | Vitamin C (mg/day | 0.160 [-0.003, 0.285]† | – | 0.098 [0.019, 0.185]* | – |
| Juice (ml/day) | Vitamin C (mg/day) and SF (μg/L) | – | 0.011 [-0.009, 0.031] | – | 0.007 [0.002, 0.023]** |
| Traditional meat (g/day) | Vitamin C (mg/day) | 0.017 [0.000, 0.114]* | – | -0.026[-0.077, -0.001]* | – |
| Traditional meat (g/day) | Vitamin C (mg/day) and SF (μg/L) | – | 0.002 [-0.001, 0.054]† | – | -0.002 [-0.011, 0.000] |

Significance level †: *p*-value < 0.10, *: *p*-value < 0.05, **: *p*-value < 0.01

*^a^*Model fit for SF (*χ2* = 194.10, *p* = 0.04, RMSEA = 0.05 [90%CI; 0.01-0.07], *p* = 0.60; CFI = 0.96; TLI = 0.95; WRMR = 1.128)

*^a^*Model fit for Hb (*χ2* = 401.85, *p <*0.0002, RMSEA = 0.06 [90%CI; 0.04-0.07], *p* = 0.20; CFI = 0.91; TLI = 0.89, WRMR = 1.30)

SF = Serum ferritin; Hb = hemoglobin; CI = confidence interval
